# Foie gras and liver regeneration: a fat dilemma

**DOI:** 10.15698/cst2018.07.144

**Published:** 2018-06-14

**Authors:** Maria Agnese Della Fazia, Giuseppe Servillo

**Affiliations:** 1 Department of Experimental Medicine, University of Perugia, Perugia, Italy.

**Keywords:** liver regeneration, Fatty Liver, ER-stress, NAFLD, SIRT1, Lipid metabolism, steatosis

## Abstract

The liver has a unique ability of regenerating after injuries or partial loss of its mass. The mechanisms responsible for liver regeneration - mostly occurring when the hepatic tissue is damaged or functionally compromised by metabolic stress - have been studied in considerable detail over the last few decades, because this phenomenon has both basic-biology and clinical relevance. More specifically, recent interest has been focusing on the widespread occurrence of abnormal nutritional habits in the Western world that result in an increased prevalence of non-alcoholic fatty liver disease (NAFLD). NAFLD is closely associated with insulin resistance and dyslipidemia, and it represents a major clinical challenge. The disease may progress to steatohepatitis with persistent inflammation and progressive liver damage, both of which will compromise regeneration under conditions of partial hepatectomy in surgical oncology or in liver transplantation procedures. Here, we analyze the impact of ER stress and SIRT1 in lipid metabolism and in fatty liver pathology, and their consequences on liver regeneration. Moreover, we discuss the fine interplay between ER stress and SIRT1 functioning when contextualized to liver regeneration. An improved understanding of the cellular and molecular intricacies contributing to liver regeneration could be of great clinical relevance in areas as diverse as obesity, metabolic syndrome and type 2 diabetes, as well as oncology and transplantation.

## INTRODUCTION

The liver has a unique ability to recover its mass after parenchymal tissue loss, a phenomenon known as liver regeneration 1,2,3. Traditionally, not only does liver regeneration represent an experimental means of elucidating the basic biology of hepatocyte proliferation, but it is also important from a clinical perspective, in that the liver can be injured by a variety of different *noxae*, including metabolic diseases, infections, toxin-related pathologies, and autoimmunity 4,5,6. Liver regeneration is likewise important in surgery, as partial hepatectomy (PH) can be performed as a means of treating hepatocellular carcinoma, and it is used in liver transplantation from live donors as well 7,8,9.

A dramatic increase in hepatic steatosis is being observed over the past few years. Non-alcoholic fatty liver disease (NAFLD) - a liver pathology closely associated with insulin resistance and the dyslipidemia-metabolic syndrome 10,11,12 - is present in a percentage as high as approximately 20-30% of any apparently healthy western populations, thus representing a clinical challenge worldwide 13. Moreover, a significant percentage of individuals with NAFLD will progress to non-alcoholic steatohepatitis (NASH) 14,15,16. Considering that as many as 25% patients with NASH will develop cirrhosis, it can be assumed that about 2% of people currently with NAFLD are expected to progress to cirrhosis 12,17. Moreover, patients with NAFLD have an increased risk of developing hepatocellular carcinoma (HCC) 18. Of note, in liver transplantation, macroscopic steatosis is associated with a higher risk of graft malfunctioning in the recipient. Because of so high a percentage of people with NAFLD, many potential donors are not eligible for donation 9.

Studies in rodents and humans have demonstrated an altered liver-regeneration pattern associated with fatty liver diseases 19,20,21,22. In all of those pathological conditions, an improved understanding of the molecular mechanisms underlying liver regeneration would be of great value from both a basic biology and clinical points of view. In this review, we will focus on the pathophysiology of fat accumulation in the liver and its consequences on liver regeneration as well as liver diseases of major relevance.

## LIVER REGENERATION IN EXPERIMENTAL MODELS

The liver presents two specific peculiarities in that it (a) maintains the homeostasis of all of the most important metabolic pathways (as regards lipids, carbohydrates and proteins) in the body 23,24,25,26 and (b) will reconstitute the original hepatic mass after injuries or partial removal of its parenchymal tissue. The two properties are interrelated, and any anomalies in metabolic homeostasis are reflected in altered liver-regeneration patterns. The capacity of the residual hepatocytes to proliferate after PH has been widely used as an experimental model of hepatocyte proliferation *in vivo* 1,2,3,27. Two important aspects of liver regeneration have been considered in detail, namely, (a) the proliferative wave whereby stable cells begin to replicate in a synchronous way, and (b) the proliferative arrest occurring when replicating cells have reconstituted the original mass.

Since the first scientific report on liver regeneration one hundred years ago 28, many efforts have been made to clarify the underlying signals and mechanisms, both cellular and molecular in nature. Over the years, many molecules have been credited with an important role in liver regeneration, but none of them have been proven to represent, singly, the pivotal factor in liver mass reconstitution 29,30,31,32. With the advent of genetically deficient knockout (KO) mice, several molecular pathways associated with those molecules have been identified as critically intercrossing at the interface of the proliferative wave and the subsequent proliferative arrest during regeneration. More often than not, the lack of a single - albeit relevant - gene in KO mice was only found to delay the process of liver regeneration, pointing to the mechanistic intricacies, and perhaps the redundancy thereof, whereby many molecules and pathways contribute to liver reconstitution 33,34,35.

Nelson Fausto proposed to assign molecules and mechanisms involved in liver regeneration to three major categories, namely, *cytokines*, *growth factors* and *metabolic networks*. In the process of liver regeneration, a variety of genes need to modify their expressions. An early set of genes is required to initiate proliferation in otherwise stable cells, which are thus forced to duplicate 36. An initial ",priming phase" is, in fact, driven primarily by cytokines, such as IL-6 and TNFα 37, and cAMP 32. By recruiting NF-κB, STAT3 and PKA, a wide number of genes become transcriptionally activated and coordinately act on residual hepatocytes 36, so to result - in turn - in transcriptional activation of the so-called early genes (such as *c-fos*, *c-myc*, and *c-jun*), which initiate the actual process of liver regeneration 38,39. Although those factors and factor-encoding genes involved in priming were previously thought as being essential to the regeneration process, studies in KO mice with PH have later shown that their respective genetic deficiencies cause only a delay in growth kinetic patterns, rather than a block in cell proliferation or an increased mortality. Therefore, the many factors associated with the priming phase do contribute to regeneration but are dispensable for overall successful completion of the process over the longer term.

The priming phase that *primes* residual hepatocytes for subsequent proliferation requires that growth factors trigger the transition from the G1 to the S phase in hepatocytes. Increased levels of hepatocyte growth factor (HGF) and epidermal growth factor (EGF) - together with the activation of the respective receptors - have long been known to be a prerequisite for proliferation 40,41,42,43. However, a variety of additional growth factors are now known to be crucially involved in this initial process 44. Remarkably, despite these changes in transcriptional programs, residual hepatocytes maintain an ability to meet the basic metabolic requirements of the whole body (in terms of glucose, amino-acid, and lipid metabolism). Even when as little as 30% (or even less) of the initial mass remains functional, metabolic homeostasis is not compromised to the benefit of the newly started proliferative process.

Notably, the many studies on liver regeneration are not to be taken as a merely mechanistic analysis of how stable cells are forced to expand; rather, they represent an extremely useful model to gain insight into basic biology issues 45,46,47,48,49 as well into the pathophysiology of liver functioning, with general regard to hepatology, transplantation, and hepatocellular carcinoma treatment 9. Many of those studies on liver regeneration have been making use of genetically deficient KO mice, with the aim of dissecting the contributions of individual genes to the regeneration process. It was only recently that one - perhaps, more clinically relevant - approach has come of age as a way to expand upon the previous information, namely, the analysis of the different patterns of liver regenerations in mice with specific dietary restrictions, such as low- or high-fat diets, low- or high-carbohydrate diets, and diets with low or high protein content 50,51.

**Figure 1:**
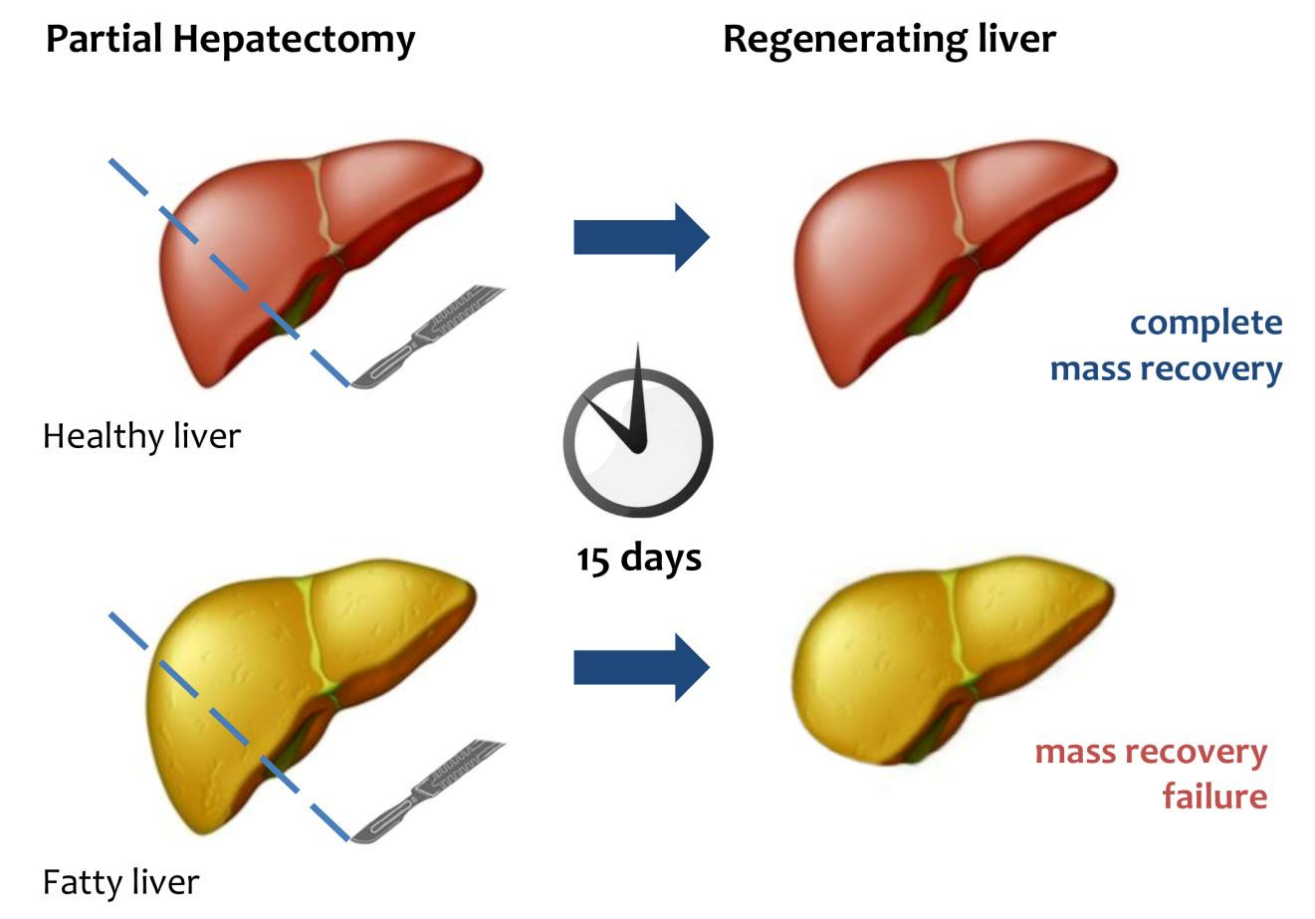
FIGURE 1: Liver regeneration failure in fatty liver. After PH, healthy liver can completely restore its mass and functional activity within 15 days. In fatty liver mass and functional recovery is compromised.

Many questions still remain unanswered, mainly as to the dynamics of liver regeneration 21,52. Among those, one of the most crucial is the functional significance of the transient steatosis observed in residual hepatocytes after PH 53,54,55,56. The issue has been widely investigated in a variety of experimental models. Over the first few hours of PH, the liver accumulates lipids, an event that is indispensable to successful liver regeneration 19,57. The major lipids being stored are free fatty acids, which are mostly mobilized from adipose tissue, with only a minority of them being derived from hepatic synthesis 58,59,60. In most studies, those lipids have been identified as being the "fuel" necessary to ignite liver regeneration 61,62,63. Additional studies - using blockade of fat accumulation either by drugs or in specific KO mice - have indicated that not only does the transient steatosis fuel the process, but it also drives the correct modality of regeneration 22. Yet, the precise mechanisms governing such a complex phenomenon have been unclear. Perhaps one consideration holds true: after PH, reconstitution of the liver mass has a priority, and the accumulated lipids need to be utilized as a source of energy to guarantee proper regeneration 64 (**Fig.1**).

## LIPID METABOLISM IN LIVER AND ER STRESS

The liver has an essential role in controlling lipid metabolism in the whole body. Cholesterol and fatty acids can be also synthesized and regulated in the liver, which coordinates the assembly of lipoproteins, mainly LDL (low density lipoprotein) and VLDL (very low density lipoprotein) 23,24,65. Under normal conditions, an overall balance between input and output of lipids in liver is maintained. Changes in this balance predispose to specific diseases such as fatty liver disease.

An important role in lipid metabolism is played by the Endoplasmic Reticulum (ER) 66,67. Several enzymes and proteins involved in lipid metabolism are present in the ER 68,69,70. Many physio-pathologic conditions will alter ER homeostasis and induce ER stress 71,72,73. A rapid request for secretory or membrane protein synthesis, as it occurs in liver regeneration, or alterations in redox or Ca^2+^ homeostasis, are conditions whereby the ER is subjected to high levels of stress 74,75. In response to many stressors, the ER triggers a well-defined process that causes the cell either to restore normal functioning or to undergo apoptosis. To accomplish the former outcome - and circumvent any potentially dangerous effects of unfolded or misfolded proteins - the ER resorts to a system of protein-quality control referred to as ER-associated degradation or ERAD 76,77,78. If ERAD, however, fails to restore protein homeostasis, the ER activates a more complex system, namely, the unfolded protein response (UPR) 79,80,81. The UPR system comprises three pathways involving transcriptional or translational regulators aimed at normalizing ER function. UPR represents a major stress pathway controlled by the chaperone 78-kDa glucose-regulated protein (GRP78) to mediate IRE1, PERK, and ATF6 signaling 82,83,84,85. Those three proteins - namely, PKR-like ER kinase (PERK), inositol-requiring enzyme-1 (IRE1), and cyclic-AMP-dependent transcription factor ATF-6α (ATF6) - are, under normal conditions, linked to the ER-chaperone GRP78. Upon ER-stress induction, GRP78 binds the unfolded or misfolded proteins as they are released in the ER lumen, thus enabling activation of the three transmembrane proteins involved in UPR. When activated, PERK homodimerizes and, in turn, phosphorylates the eukaryotic initiation factor 2α (eIF2α), which prevents the 80S ribosome assembly, leading to the arrest of protein synthesis 82,86.

Even though phosphorylation of eIF2α arrests protein synthesis it allows the translation of selected protein including the transcription factor ATF4, which is overexpressed during ER stress and regulates expression of a number of genes responsible for amino-acid metabolism and apoptosis, including C/EBP homologous protein (CHOP) 87. Notably, ATF4 controls the expression of GADD34, which in turn plays an important role in dephosphorylating eIF2α, thus activating a key feedback mechanism to restore protein synthesis 88. Of note, eIF2a can be phosphorylated not only via activation of the UPR system, but also by other kinases involved in the stress response 89.

The ER stress-driven UPR system makes use of another transmembrane protein, IRE1. IRE1 is a multifunctional protein, capable of kinase and ribonuclease activities 90. The RNAse activity allows the generation of a splicing form of the X-box-binding protein 1 (XBP1), which acquires an ability to transcribe ER-chaperons, proteins of the ERAD system, and proteins involved in fatty-acid metabolism 91,92,93.

ATF6 is a further UPR-pathway protein activated by ER stress. ATF6 binds GRP78 in the ER, yet in response to stressors, it is released from GRP78 and migrates to the Golgi, where it is cleaved and moves to the nucleus. ATF6 works as a transcription factor coordinating the expression of different components of the UPR system (i.e., GRP78), ER-chaperones, XBP1, the pro-apoptotic factor CHOP, as well as other components of ER-stress response 94,95.

**Figure 2:**
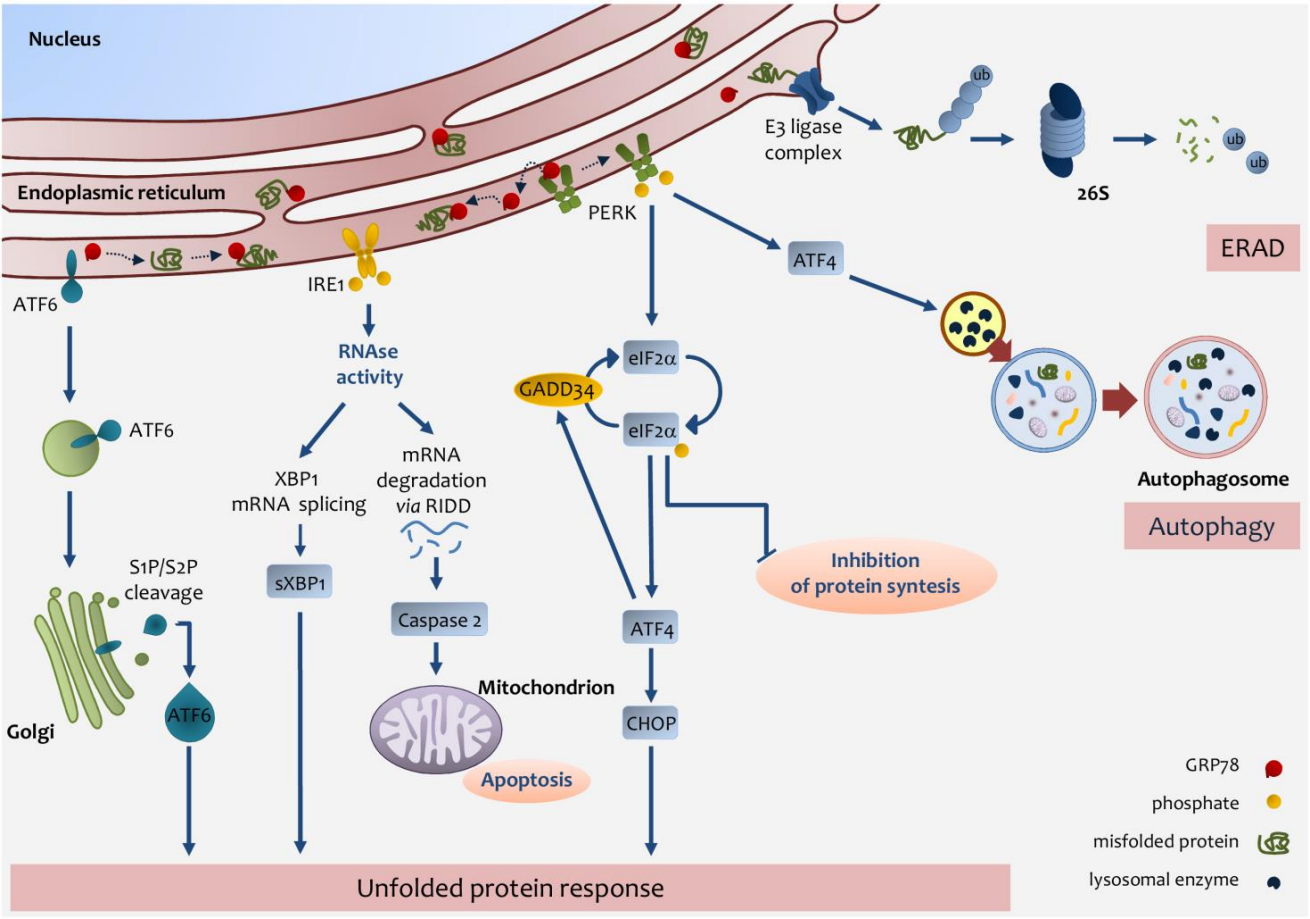
FIGURE 2: Endoplasmic reticulum (ER) stress pathways. ER stress occurs through the accumulation of misfolded proteins in the ER lumen. The role of the UPR is to re-establish ER homeostasis by reducing *de novo *protein synthesis and improving the folding and clearance capacity of the ER. The first mechanism against ER stress is ERAD, by which misfolded proteins are translocated back to the cytosol, ubiquitylated and degraded by the proteasome. When the level of misfolded proteins is too high, the cell activates UPR consisting in the activation of three transmembrane pathways. The main effectors are PERK, IRE1 and ATF6. Effectors activation is initiated by the removal of GRP78 allowing the translocation of the latter from the ER membrane to the ER lumen where it associates with unfolded proteins. eIF2α phosphorylation inhibits protein translation except for ATF4 which induces the expression of factors involved in antioxidant defence, amino acid metabolism, autophagy and apoptosis, such as CHOP. ATF4 also induces the expression of GADD34, which expression enables the dephosphorylation of eIF2α and the re-initiation of translation. PERK-mediated induction of ATF4, can also promote the expression of key autophagy-related proteins, which are needed for autophagosome formation. Once activated, IRE1 promotes the splicing of *XBP1 *mRNA, which is then translated into the active sXBP1, which transactivates the expression of components related to protein folding, ERAD and protein quality control. IRE1 also promotes the degradation of RNAs localized in the ER vicinity by regulated IRE1-dependent decay (RIDD), which induces caspase-2 and mitochondrial apoptosis. Upon GRP78 release, ATF6 is transferred to the Golgi apparatus as inactive precursors and cleaved by membrane-bound site-1 (S1P) and site-2 (S2P) proteases into an active form, which induces the expression of chaperones and UPR components. IRE1-mediated activation of XBP1 as well as ATF6 activation induces the expression of chaperones and UPR components.

Finally, the cell to tackle against stressors has developed, ERAD, UPR, or autophagy, but in the case of failure in adaptive response the UPR sensors addressed the cell towards apoptosis 96 (**Fig. 2**).

## ER STRESS AND LIVER REGENERATION

Because of the role of liver in controlling lipid homeostasis, it is clear that the UPR system is considered to be pivotal in the response to a number of stressors acting on hepatocytes. Chemical or naturally occurring toxicants act on hepatocytes and changes in cellular status - such as nutritional and proliferative changes or rapid functional requests - are accomplished via transient ER stress and UPR activation 97,98,99,100. Indeed, proliferation of residual hepatocytes in the onset of liver regeneration needs abundant protein synthesis, which increases protein folding demand within the ER. At present, UPR function is considered a major component of liver regeneration. The analysis of pivotal players in UPR shows a triggering event during liver regeneration. Physiological response involves a rapid increase and activation of ER-stress genes after PH. IRE1a pathway, PERK, pIF2a and CHOP increase their expression in the first hours after partial hepatectomy 101,102,103.In particular, after a 70% PH, residual hepatocytes undergo changes that drive ER stress 20,104. As a matter of fact, the residual tissue (30%) needs to preserve the overall homeostasis in the body while engaged in reconstituting liver mass. The latter event requires that residual hepatocytes are able to synthesize and assemble all the cellular components needed for regeneration, which calls for the onset of a transient ER-stress status, the hallmark of which is temporary steatosis, reversed only by the time of regeneration completion 20,53,64.

Such a bidirectional relationship would imply that an excess of dietary lipid intake resulting in steatosis might determine a status of chronic activation of the ER-stress response. Experiments in obese rodents have indeed provided evidence for the persistent activation of the UPR system, a condition shared clinically by patients with severe steatosis, NAFLD, or NASH 105,106,107. The major mechanism responsible for the association between steatosis and activation of the UPR system seems to be related to changes in membrane fluidity and consequent loss of Ca^2+^ homeostasis. Changes in membrane composition and fluidity have been described in obese mice that display an altered ratio between the two main membrane phospholipids, phosphatidylcholine and phosphatidylethanolamine 108. An altered ratio between the two phospholipids leads to ER membrane modification, which, in turn, affects Ca^2+^ homeostasis. As demonstrated by a series of elegant experiments, a decrease in Ca^2+ ^causes remarkable stress in the ER via reduction in the Ca^2+^-dependent chaperone component. Overall, these data substantiate the role of lipids as one of the most important ER stressor 109,110.

Many authors have identified ER stress as being, itself, the primary cause of the *de novo* synthesis of lipids either under conditions of steatosis-associated insulin resistance or of chronic ER stress-induced proteolytic cleavage of SREBP-1c, which translocates to the nucleus and activates a cohort of genes involved in lipogenesis 111. Therefore, the outcome of chronic activation of ER stress by lipids is steatosis and subsequent onset of NAFLD. This is determined by lipid accumulation in the liver and ER-stress activation, which plays an important role *per se* in the pathogenesis of NAFLD, mainly by direct control of *de novo* lipogenesis and by mitochondria modifications 112,113. On the long term, chronic ER stress recruits additional mechanisms in the liver, including a reduction in VLDL secretion or a change in the insulin response, which exacerbates the condition 114. Such a prolonged condition of the liver causes increased oxidative stress accompanied by inflammation and apoptosis, eventually resulting in the onset of NASH. Overall, chronic activation of ER stress is a pathological condition that determines a severe impairment in liver functioning 104,115.

The regenerative ability of the liver under ER stress is altered and even in absence of severe steatosis or NAFLD, the residual hepatocytes have a delay in their ability to proliferate properly 116,117,118. Recent studies in mice fed a high-fat diet for 10 weeks to induce steatosis showed that the classical genes involved in ER stress - with the exclusion of sXBP-1 - were not overexpressed. After PH, a delay in the proliferation of residual hepatocytes was observed in mice fed the high-fat diet; higher a ctivation of ER-stress genes, such as GRP78, IRE1a, ATF6, PERK, sXBP-1 and CHOP, was detected 20. Moreover, persistent fat accumulation was present in mice fed the diet. Mice genetically deficient for the 3 β-hydroxysterol Δ14-reductase (TM7SF2)-encoding gene (TM7SF2 KO mice, which lack the ER enzyme involved in cholesterol synthesis) showed abnormal

activation of the ER-stress response. PH in TM7SF2 KO mice resulted in an impaired proliferation relative to wild-type counterparts, with a delay in the cell-cycle G1/S transition and early activation of GRP78. Furthermore, the KO mice accumulated an anomalous amount of hepatic triglycerides until 60 hours of PH. Unusual activation of p53 and persistently elevated levels of p21 were observed in the KO mice during liver regeneration. Because of the control of p21 on CHOP, it is conceivable that elevated level of p21 in those mice is related to the activation of the ER-stress response by CHOP 104. The analysis of liver regeneration after PH in different KO mice, in which any genes involved in ER-stress are deleted, shows regeneration abnormalities with altered proliferation of the residual hepatocytes. Impairment in regenerative process has been detected in mice with IRE1a hepatocyte-specific deletion 101. Moreover, the ER stress has been defined as an important cellular process in liver regeneration under Ischemia/Reperfusion Injury 119 as demonstrated in CHOP KO mice 120.

Whether a perturbation in the ER-stress response is directly responsible for the delay in liver regeneration, or an altered ER-stress response sustains fat accumulation responsible for the impaired liver regeneration, is still to be defined.

## SIRTUINS AND LIPID METABOLISM

Many studies have been describing an important role for sirtuins in the metabolism of liver lipids. Sirtuins are a family of proteins that share a number of functions and which conserve NAD^+^-dependent histone and protein deacetylase functions. In mammals there are seven sirtuins with different cellular functions ranging from energy metabolism, cellular stress resistance, genomic stability to aging and tumorigenesis 121,122. Each Sirtuin has distinct functions and subcellular localizations. SIRT1 and SIRT2 are in both the nucleus and the cytosol, SIRT3, SIRT4, and SIRT5 are in the mitochondria and SIRT6 and SIRT7 are localized in the nucleus 123. Among them, SIRT1, SIRT3, SIRT6 and SIRT7 have been discovered as involved in fat liver metabolism 124,125,126,127,128.

The silent information regulator 1 (SIRT1) represents one of the best-characterized members of the mammalian sirtuin family of NAD-dependent histone deacetylases. SIRT1 is a nutrient sensor and has a crucial role in the control of ageing in different organisms 129. Moreover, SIRT1 has a pivotal role in the control of normal liver function in mammals, participating in the regulation of metabolic processes such as gluconeogenesis, fatty acid β-oxidation and cholesterol flux 122.

Of particular interest, SIRT1 has been known as an important regulator of circadian rhythms 130,131,132,133. SIRT1 directs circadian oscillation in hepatocytes through rhythmic deacetylation of histone H3 at the promoter of clock-controlled genes. Moreover, SIRT1 controls deacetylation of important circadian regulators such as BMAL1 and PER2 130,132. Deficiency of liver-specific SIRT1 - using SIRT1-KO (Sirt^LKO^) mice - as well as pharmacological modulation of SIRT1 expression lead to major changes in hepatic circadian gene expression and lipid metabolism 134,135.

SIRT1 crucially acts as a sensor in metabolic and energy control of the cell 136,137. SIRT1 activity is mediated by NAD^+^ levels 138. Because the liver is the major organ controlling homeostasis, it is clear that perturbations driven by high-fat or high-calorie diets, alcohol or drugs altering NAD^+^ levels will all affect SIRT1 transcriptional activity 124,139,140,1141. Several participants in liver homeostasis are regulated by SIRT1, most of them being transcription factors with a role in lipid metabolism, such as the peroxisome proliferator-activated receptor-α (PPARα), peroxisome proliferator-activated receptor-γ coactivator-1α (PGC-1α) 142, carbohydrate response element binding protein (ChREBP), sterol regulatory element binding protein-1c (SREBP-1) 143, and NF-κB 144,145. The control of SIRT1 occurs *de facto* via deacetylation of transcription factors involved in lipid metabolism 146. SREBP-1c and ChREBP deacetylation is accompanied by an arrested transcription of downstream genes, leading to lipogenesis. PPARα and PGC-1α deacetylation promotes FA-β-oxidation. Owing to the role of SIRT1 in controlling liver lipid metabolism, many studies have been conducted in Sirt^LKO ^mice 147. In particular, it was found that Sirt^LKO^ mice, fed a normal diet, develop enhanced fat accumulation in their livers over their entire lifespans, indicating a clear participation of SIRT1 in the onset of hepatic steatosis 147,148,149,150. Indeed, more than with steatosis alone, the overall clinical picture is consistent with an underlying inflammatory condition and increased ROS production, both of which accompany the hyperglycemia and insulin resistance typically driven by steatosis in Sirt^LKO ^mice 151.

Interestingly, PH performed in Sirt^LKO ^mice results in impaired liver regeneration, with a clear delay in the G1/S transition, deregulation of cyclins and related CDKs and delayed regeneration. Moreover, the regenerating liver from Sirt^LKO^ mice shows unusual accumulation of lipids, with increased levels of TAGs (triacylglycerol), NEFA (non-esterified fatty acid) and cholesterol, thus confirming the crucial role of SIRT1 in regulating fat in liver under regeneration. Notably, under the same conditions, liver regeneration is characterized by downregulation of PPARα-related genes, thus substantiating a role for SIRT1 in controlling PPARα and, in turn, fatty acid β-oxidation 152. These findings are in agreement with previous results in PPARα KO mice, in which a delay occurs in liver regeneration relative to control counterparts. Overall it is clear that, by balancing fat composition, SIRT1 has an important role in liver regeneration 153.

The recent finding of an interaction between SIRT1 and ER stress in the liver has been receiving much attention 154. Whether lack of SIRT1 after PH - leading to hepatic fat composition - drives ER-stress activation or, conversely, it is the lack of SIRT1 - resulting in unrestrained ER stress - that matters so much, remains to be explained. On the one hand, many studies have demonstrated that SIRT1 overexpression alleviates ER stress; SIRT1 overexpression in obese mice is accompanied by a reduction in ER stress and steatosis 155,156,157. On the other hand, recent studies have credited SIRT1 with an ability to control the activation of ER stress, by deacetyling the fundamental transcription factor XBP1, and by controlling eIF2α, which triggers ER stress 158. Overall, the interplay between ER stress and SIRT1 is intricate and complex, but recent reports have been providing new insight into this relationship, mostly by focusing on the role of ER stress in controlling SIRT1. *In vivo* and *in vitro* experiments showed that, by activating the PI3K-AKT-GSK3b pathway, ER stress would induce expression of SIRT1 159.

**Figure 3:**
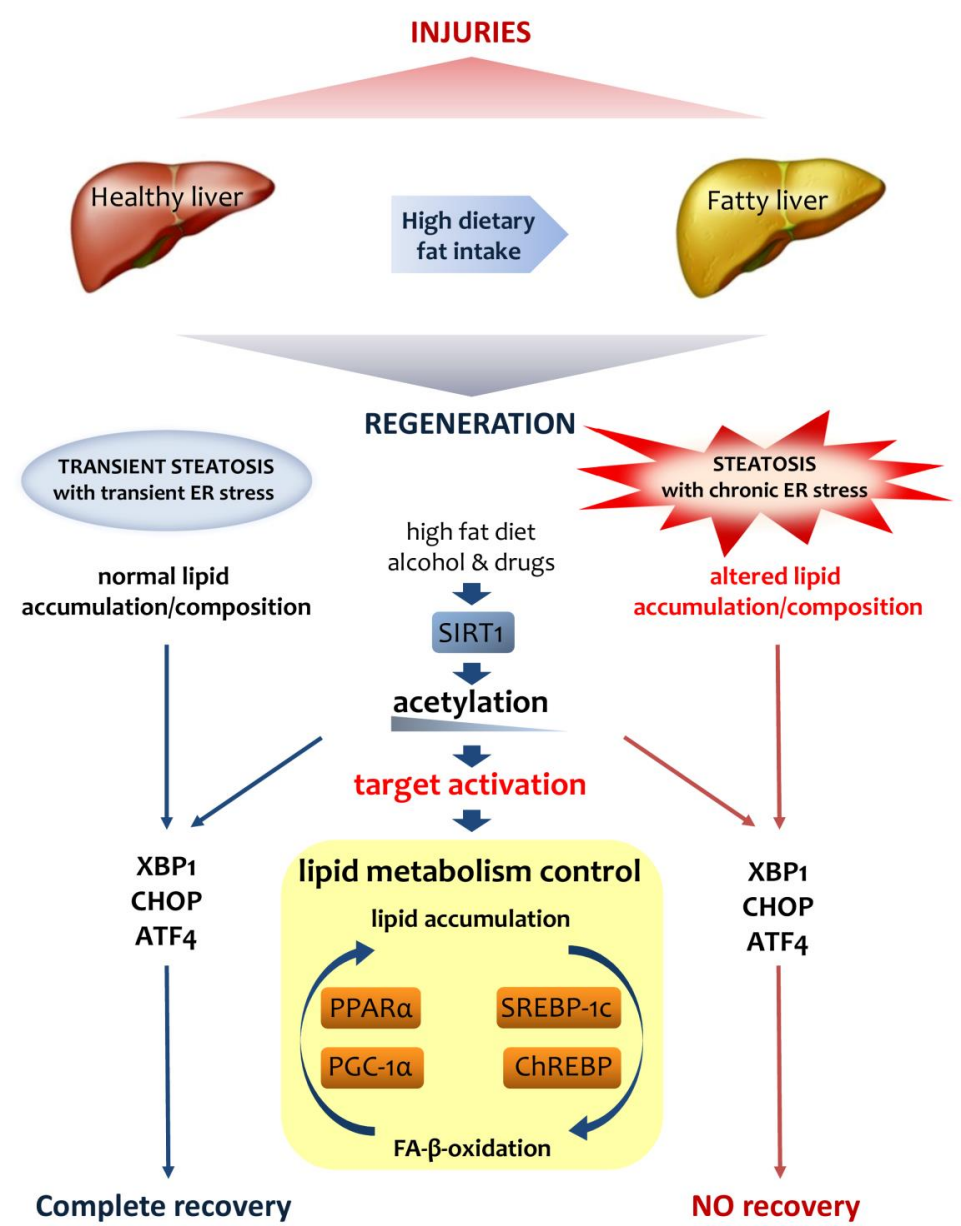
FIGURE 3: Lipid metabolism is crucial for liver regeneration. Fatty liver has an altered metabolism in lipids that affects its ability to regenerate after various injuries. Lipid accumulation during liver regeneration induces a transient steatosis with transient ER stress that is functional to proper proliferation of hepatocytes. On the contrary, the chronic steatosis and ER stress in fatty liver result in an altered lipid asset that could be addressed as the origin of the failure of regeneration. SIRT1, as a major player in lipid metabolism control, through its deacetylase activity on H3-histone at gene promoters, controls the activation of various UPR genes such as XPB1, CHOP and ATF4.

Interestingly, SIRT1 and ER stress share another important function in the liver. SIRT1 is a pivotal regulator of circadian rhythms in the liver, where it has an ability to deacetylate histone H3 in a rhythmic manner, which, in turn, controls the promoters of clock-controlled genes as well as BMAL1 and PER2 130,132. Altered SIRT1 activity in the liver is reflected in an altered expression of circadian genes and lipid metabolism 134,135. Importantly, the circadian clock coordinates the activation of important genes regulating ER stress in the liver; an altered circadian rhythm perturbs ER stress and, consequently, lipid metabolism 160,161,162. Increasing evidence is available regarding the relationship between lipid metabolism and circadian clock, with a focus on timing of food intake, liver stress and liver pathology (**Fig.3**).

SIRT3 is a mitochondrial protein, which plays an important role in the control of carbohydrate metabolism, ketogenesis, β-oxidation, and amino-acid metabolism 163. Chronic high fat diet and obesity reduced SIRT3 activity, which in turn is associated with fatty liver. Indeed, analysis of SIRT3-KO mice shows no metabolic difference respect to the WT mice even if differences have been found in the mitochondrial acetylation proteins 164. Hyperacetylation of mitochondrial protein found in SIRT3-KO mice accelerates the development of metabolic syndrome with steatohepatitis 165. Recently, a study on pancreatic b-cells showed that SIRT3 is involved in the regulation of ER-stress down-regulating the gene expression of protein of ER-stress 165. SIRT6 and SIRT7 have been investigated to analyze their role in NAFLD. The analysis of Hepatocyte-Specific SIRT6-KO mice have revealed a predisposition to NAFLD when fed with a high-fat and high-fructose (HFHF) diet for 16 weeks. HFHF-diet induced in SIRT6-KO mice an increased in hepatic steatosis and inflammation with liver fibrosis and oxidative stress 166.

SIRT7 is a nuclear protein, highly expressed in the liver that regulates metabolic homeostasis 167. It has been demonstrated that SIRT7 specific liver deficient mice develop steatosis with elevated expression of inflammatory markers indicating a turning towards NASH. Moreover, it has been revealed that in SIRT7 specific liver deficient mice the liver steatosis is not accompanied with obesity. Notably, it has been discovered that in liver SIRT7 regulates ER-stress by repressing Myc activity 168.

Nevertheless, a clear picture of the complex relationship between Sirtuins and ER stress is still lacking (**Table 1**).

**Table 1:** TABLE 1. Summary of effects of Non-alcoholic fatty liver disease (NAFLD) in various models on liver regeneration.

**Experimental model**	**Factors that regulates its expression/level**	**Effect**	**Reference**
High-Fat Diet	IκBα, NF-κB, Cyclin D1 and TNF-α	Development of increased body weight	21
		Steatotic livers to increased injury through IκBα overexpression and subsequent NF-κB inhibition	
		Impaired liver regeneration	
db/db mice	VEGF and EGFR	Altered angiogenesis	57 169
		Impaired liver re generation	
ob/ob mice CCl4	PEPCK, Cyclin D1 and TNF	Altered rate-limiting enzyme for hepatic gluconeogenesis	170
		Delay of expression of Cyclin D1 was insufficient to drive the cells into S-phase	
		Defective TNF release by hepatic Kupffer cells and circulating macrophages induces the impaired hepatocyte proliferation	
ob/ob mice	TNF, IL-6, STAT-3 and p21	Impaired liver regeneration	19,171,172
dietary fructose compared to dietary fat	CPT-1, PPAR-α, AMPK and Cyp2E1	Steatosis	173
		Impaired liver regeneration in fatty liver is related to the cause, but not necessarily to the degree, of hepatic steatosis.	
western diet (WD)	TNFR-1, CD95/Fas, Noxa, Bcl2, Bcl-xl, Mcl-1 and HGF	Partial hepatectomy in steatotic liver doesn’t affect hepatocyte apoptosis, despite DR upregulation	174
		WD-induced steatosis enhances liver cell proliferation, which is accompanied by increased HGF and leptin signaling as well as Erk1/2 phosphorylation	
Zucker (fa/fa) rats	PCNA and Cyclin E	Steatosis per se does not impair liver regeneration.	156
		The reduced liver regeneration observed in obese Zucker rats may not be due to fatty infiltration itself but to other factors such as leptin receptor dysfunction.	

## CONCLUSIONS

Liver regeneration represents a unique model for studying proliferation under conditions in which stable cells are induced to proliferate to rectify any parenchymal loss. Yet, the model is not simply meant to prove insight into a biological and legendary curiosity - the legend of Prometheus, chained to a rock, where an eagle ate his liver during the day, and the liver was regenerated during the night due to Prometheus' immortality. The knowledge, indeed, of the unique and complex mechanisms that determine liver regeneration might be of great help to a better understanding of liver pathology and to improved therapeutic and surgical interventions.

Broad panoply of events influences the ability of liver to regenerate, and recently fat liver accumulation has been receiving much attention, because of the high incidence of steatosis in humans. Macroscopic or severe steatosis has a negative impact on regeneration of the liver in that it opposes proper regeneration in patients after surgical removal of neoplasia or in liver grafts. Moreover, because of the high incidence of NAFLD, steatosis represents a significant limitation to the availability of potential liver donors.

The knowledge of the molecular mechanisms governing liver regeneration and their impact on human liver pathology is becoming increasingly relevant in the clinical context. A better comprehension of the factors responsible for fat accumulation and their potential role in liver regeneration might permit the identification of novel druggable targets in hepatology 175.

## Abbreviations

ER: Endoplasmic Reticulum
ERAD: ER-associated degradation
NAFLD: non-alcoholic fatty liver disease
NASH: non-alcoholic steatohepatitis
PH: partial hepatectomy
UPR: unfolded protein response
